# FAK tyrosine phosphorylation is regulated by AMPK and controls metabolism in human skeletal muscle

**DOI:** 10.1007/s00125-017-4451-8

**Published:** 2017-10-11

**Authors:** David G. Lassiter, Carolina Nylén, Rasmus J. O. Sjögren, Alexander V. Chibalin, Harriet Wallberg-Henriksson, Erik Näslund, Anna Krook, Juleen R. Zierath

**Affiliations:** 1 Department of Molecular Medicine and Surgery, Section for Integrative Physiology, Karolinska Institutet, von Eulers väg 4a, IV, SE-171 65 Stockholm, Sweden; 2 Department of Physiology and Pharmacology, Integrative Physiology, Karolinska Institutet, Stockholm, Sweden; 3 Division of Surgery, Department of Clinical Sciences, Danderyd Hospital, Karolinska Institutet, Stockholm, Sweden; 4 Section of Integrative Physiology, The Novo Nordisk Foundation Center for Basic Metabolic Research, Faculty of Health and Medical Science, University of Copenhagen, Copenhagen, Denmark

**Keywords:** AICAR, AMPK, Focal adhesion kinase, Gene silencing, Glycogen synthesis, Insulin, Lipid oxidation, Metabolic flexibility, Open-muscle biopsy, Skeletal muscle

## Abstract

**Aims/hypothesis:**

Insulin-mediated signals and AMP-activated protein kinase (AMPK)-mediated signals are activated in response to physiological conditions that represent energy abundance and shortage, respectively. Focal adhesion kinase (FAK) is implicated in insulin signalling and cancer progression in various non-muscle cell types and plays a regulatory role during skeletal muscle differentiation. The role of FAK in skeletal muscle in relation to insulin stimulation or AMPK activation is unknown. We examined the effects of insulin or AMPK activation on FAK phosphorylation in human skeletal muscle and the direct role of FAK on glucose and lipid metabolism. We hypothesised that insulin treatment and AMPK activation would have opposing effects on FAK phosphorylation and that gene silencing of FAK would alter metabolism.

**Methods:**

Human muscle was treated with insulin or the AMPK-activating compound 5-aminoimadazole-4-carboxamide ribonucleotide (AICAR) to determine FAK phosphorylation and glucose transport. Primary human skeletal muscle cells were used to study the effects of insulin or AICAR treatment on FAK signalling during serum starvation, as well as to determine the metabolic consequences of silencing the FAK gene, *PTK2*.

**Results:**

AMPK activation reduced tyrosine phosphorylation of FAK in skeletal muscle. AICAR reduced p-FAK^Y397^ in isolated human skeletal muscle and cultured myotubes. Insulin stimulation did not alter FAK phosphorylation. Serum starvation increased AMPK activation, as demonstrated by increased p-ACC^S222^, concomitant with reduced p-FAK^Y397^. FAK signalling was reduced owing to serum starvation and AICAR treatment as demonstrated by reduced p-paxillin^Y118^. Silencing *PTK2* in primary human skeletal muscle cells increased palmitate oxidation and reduced glycogen synthesis.

**Conclusions/interpretation:**

AMPK regulates FAK signalling in skeletal muscle. Moreover, siRNA-mediated FAK knockdown enhances lipid oxidation while impairing glycogen synthesis in skeletal muscle. Further exploration of the interaction between AMPK and FAK may lead to novel therapeutic strategies for diabetes and other chronic conditions associated with an altered metabolic homeostasis.

**Electronic supplementary material:**

The online version of this article (10.1007/s00125-017-4451-8) contains peer-reviewed but unedited supplementary material, which is available to authorised users.

## Introduction

Skeletal muscle is a highly malleable tissue, capable of remodelling physical and biochemical properties to meet changes in cellular and whole-body metabolic demands [1]. Type 2 diabetes is characterised by multiple defects in skeletal muscle including insulin resistance, defective oxidative metabolism, altered mitochondrial function and loss of muscle mass [2]. Defects in insulin action in skeletal muscle are also noted in non-diabetic first-degree relatives of people with type 2 diabetes [3], indicating that insulin resistance is an early event in the pathogenesis of type 2 diabetes. Delineation of signalling pathways emanating from the insulin receptor and AMP-activated protein kinase (AMPK), two major processes controlling glucose and energy homeostasis [4], is central in the efforts to resolve type 2 diabetes pathogenesis. How these signalling networks are kept in homeostatic balance is not completely clear.

Under physiological conditions, AMPK signalling is activated during intracellular energy deprivation, while insulin signalling occurs during systemic energy surplus [5, 6]. Consequently, AMPK and insulin signalling can have opposing roles, with the former prioritising energy utilisation and inhibiting growth and the latter prioritising energy storage and promoting growth [7]. To achieve energy balance, AMPK and insulin signalling can also converge at common nodes to coordinate metabolic responses. Examples of common nodes shared by these pathways include two Rab GTPase-activating proteins, TBC1 domain family member 1 (TBC1D1) and TBC1 domain family member 4 (TBC1D4; also known as Akt substrate of 160 kDa, AS160). These proteins respond to AMPK activation and insulin stimulation and are involved in the regulation of glucose transport [2]. Elucidation of other molecular points of crosstalk between AMPK and insulin signalling may reveal how these pathways are coordinated to meet the energy demands of the cell.

Focal adhesion kinase (FAK) may play a role in integrating insulin signalling and energy-sensing signals within human skeletal muscle. FAK is a mechanosensitive/exercise-responsive protein that plays a role in skeletal muscle morphology, metabolism and insulin sensitivity [8–12]. Activation of FAK is carried out first via autophosphorylation at Y397 and subsequently via phosphorylation at Y576/Y577 [11]. The proper timing of FAK activation via autophosphorylation at Y397 is essential for normal myoblast differentiation [13] and skeletal muscle hypertrophy after hindlimb suspension in rodents [14]. During ageing, impaired FAK signalling is associated with functional decline in the regenerative potential of skeletal muscle stem cells [15]. Several lines of evidence link AMPK and FAK signalling. In immortalised vascular smooth muscle cells from rats, AMPK and FAK are counter-regulated by an α-glucosidase inhibitor [16]. In addition, in HepG2 cells (a human liver cancer cell line), overexpression of the AMPK-related kinase sucrose non-fermenting AMPK-related kinase (SNARK) reduces FAK phosphorylation [17]. Mutant forms of FAK impair insulin signalling in HepG2 cells [18] and tail-vein injection of siRNA against the FAK gene (*PTK2*) leads to insulin resistance, concomitant with reduced protein kinase B (PKB, also known as Akt) phosphorylation in mouse models of diabetes [9]. Furthermore, FAK plays a central role in maintaining cell survival and insulin sensitivity in mouse adipose tissue [19]. Because FAK plays a role in contractile- and insulin-responsive signals in rodent muscle, it is a candidate protein to mediate energy balance due to AMPK and insulin signalling in human skeletal muscle.

Here we determined the role of FAK in human skeletal muscle as it relates to insulin stimulation and AMPK activation. We hypothesised that insulin treatment and AMPK activation would have opposing effects on FAK phosphorylation. As a secondary objective, we used siRNA-mediated gene silencing to test the hypothesis that FAK plays a role in glucose and lipid metabolism in human skeletal muscle cells.

## Methods

### Ethics statement

Informed consent was obtained from all participants. The experimental procedures were approved under the license number 2012/1955-31/1 by the local ethical committee and were conducted according to the Declaration of Helsinki.

### Study participants

Eleven healthy men from the Stockholm area volunteered for this study. The clinical characteristics of the study cohort are presented in Table 1. Participants reported to Danderyd Hospital (Stockholm, Sweden) in the morning following a 12 h fast and a 24 h abstention from physical exercise.

**Table 1:** Characteristics of the study participants

Clinical feature	Mean±SEM
Age, years	50.6 ± 2.4
Height, cm	179.9 ± 2.4
Weight, kg	81.4 ± 3.3
BMI, kg/m^2^	25.1 ± 0.6
Waist-to-hip ratio	0.89 ± 0.01
Systolic blood pressure, mmHg	125.0 ± 3.9
Diastolic blood pressure, mmHg	79.5 ± 1.8
Fasting plasma glucose, mmol/l	5.3 ± 0.1
Fasting insulin, pmol/l	49.9 ± 7.9
HbA_1c_, %	5.3 ± 0.1
HbA_1c_, mmol/mol	34.5 ± 0.9
HDL-cholesterol, mmol/l	1.3 ± 0.1
LDL-cholesterol, mmol/l	3.9 ± 0.1
Triacylglycerol, mmol/l	0.9 ± 0.2
Total cholesterol, mmol/l	5.7 ± 0.1

Data are for *n* = 11 men

### Open-muscle biopsy procedure, glucose transport and intracellular signalling

Vastus lateralis muscle was obtained using an open-muscle biopsy technique as described previously [20]. Skeletal muscle strips were dissected from the biopsy specimen, mounted on Plexiglass clamps and incubated for 30 min in a recovery buffer (oxygenated Krebs–Henseleit buffer containing 5 mmol/l HEPES, 0.1% wt/vol. bovine serum albumin, 15 mmol/l mannitol, 5 mmol/l glucose). Muscle strips were subsequently incubated for 20 min in the absence or presence of 120 nmol/l insulin (Actrapid, Novo Nordisk, Bagsværd, Denmark) and/or 2 mmol/l 5-aminoimadazole-4-carboxamide ribonucleotide (AICAR) (Toronto Research Chemicals, Toronto, ON, Canada). Insulin and/or AICAR were absent or present at the same concentrations in all subsequent buffers. Muscle strips were incubated for 10 min in glucose-free rinse buffer containing 20 mmol/l mannitol and subsequently for 20 min in buffer containing 15 mmol/l mannitol, 5 mmol/l 3-*O*-methylglucose and 14,800 Bq/ml ^14^C-labelled mannitol and 148,000 Bq/ml of ^3^H-labelled 3-*O*-methylglucose. Thereafter, muscle strips were trimmed of connective tissue and frozen with a clamp pre-cooled in liquid nitrogen. Methanol or DMSO was present at concentrations of 0.05% or 0.1% vol./vol., respectively, in all buffers except for the first; no significant effects were detected due to these solvents and data were pooled in all analyses.

Muscle strips were pulverised in a lysis buffer (10% vol./vol. glycerol, 1% vol./vol. Triton X-100, 137 mmol/l NaCl, 20 mmol/l TRIS at pH 7.8, 10 mmol/l NaF, 2.7 mmol/l KCl, 1 mmol/l MgCl_2_, 1 mmol/l EDTA, 0.5 mmol/l NaVO_3_, 0.2 mmol/l phenylmethane sulfonyl fluoride, and 1:100 protease inhibitor cocktail set 1 [Merck Millipore, Billerica, MA, USA]). Lysates were centrifuged and supernatants were separated from the insoluble component. The protein content of the supernatants was assessed by a Pierce BCA protein assay kit (Thermo Fisher Scientific, Waltham, MA, USA). A portion of the lysates was used to determine 3-*O*-methylglucose transport as described [21] and the rest of the sample was used to analyse intracellular signalling by western blot as described [22]. Protein lysates were diluted in Laemmli buffer, subjected to SDS-PAGE, transferred to Immobilon-P polyvinylidene fluoride membranes (Merck Millipore), washed in TRIS-buffered saline (TBS) with Tween-20 (TBST), blocked in 7.5% wt/vol. non-fat dry milk and incubated overnight at 4°C with primary antibodies (1:1,000) in TBS containing 0.1% wt/vol. bovine serum albumin and 0.1% wt/vol. NaN_3_. Membranes were incubated with horseradish peroxidase-conjugated secondary antibodies (Thermo Fisher Scientific, 1:25,000) in TBST with 4% wt/vol. non-fat dry milk and subsequently with extended chemiluminescence reagents (GE Healthcare, Little Chalfont, UK). Primary antibodies are listed in ESM Table 1.

### FAK signalling in primary human skeletal muscle cells

Primary skeletal muscle cell cultures were established, grown and differentiated from satellite cells derived from vastus lateralis skeletal muscle biopsies taken from people with normal glucose tolerance as described [22]. Cells were grown in ‘growth media’, differentiated for 4–8 days in ‘differentiation media’ and differentiation was completed by maintaining cells in ‘post-differentiation media’ for 4–8 days prior to experimental treatments. The exact formulations of these media have been described previously [22].

To examine the effects of serum starvation on FAK signalling, myotubes were incubated for 3–6 h in media with or without serum. To explore the effects of insulin and AICAR treatment on FAK signalling, other myotubes were treated with 120 nmol/l insulin or 2 mmol/l AICAR in serum-free media. Cells were rinsed twice in ice-cold PBS and then frozen at −20°C until subsequent western blot analysis. All results were compared with those obtained from untreated myotubes harvested at 0 h.

### *PTK2* gene silencing, palmitate oxidation, glycogen synthesis and intracellular signalling in primary human skeletal muscle cells

Differentiated cells were transfected twice, separated by 48 h, using Lipofectamine RNAiMAX Transfection Reagent along with 10 nmol/l of a non-targeting negative control siRNA or siRNA directed against *PTK2* (silencer select Negative control No.2, no. 4390847, or validated silencer select siRNA s11485, respectively; Thermo Fisher Scientific). To determine gene-silencing efficiency, mRNA was harvested from cells using the E.Z.N.A. Total RNA Kit 1 (Omega Bio-tek, Norcross, GA, USA). Reverse transcription and quantitative PCR were carried out using MultiScribe Reverse Transcriptase and Fast SYBR Green Master Mix, respectively (Thermo Fisher Scientific). mRNA expression of *PTK2* and reference genes (*PPIB*, *TBP*, *B2M* and *TFRC*) was assessed using self-designed oligonucleotides (Sigma-Aldrich, St Louis, MO, USA). Oligonucleotide sequences are listed in ESM Table 2.

To assess the effects of *PTK2* on lipid oxidation, myotubes were exposed to serum-free post-differentiation media containing 0.025 mmol/l palmitate and incubated in the absence or presence of 2 mmol/l AICAR for 6 h. A fraction of the palmitate (approximately 1:300) was radioactively labelled (9,10-[^3^H]palmitate, NET043005MC; PerkinElmer, Waltham, MA, USA). Thereafter, media was collected and myotubes were lysed in 0.03% wt/vol. SDS. The protein content of the cellular lysate was assessed by a colorimetric assay (Protein Assay Dye Reagent no. 5000006; Bio-Rad, Hercules, CA, USA). The radioactivity of ^3^H-labelled water in the media was assessed by scintillation counting after isolation from non-oxidised radioactive palmitate using centrifugation with activated charcoal. Palmitate oxidation was normalised to protein content. To assess the effect of *PTK2* silencing on intracellular signalling, cells were incubated for 1 h in the absence or presence of 120 nmol/l insulin or 2 mmol/l AICAR ~ 48 h after the final transfection. Cells were rinsed twice in ice-cold PBS and then frozen at −20°C until subsequent western blot analysis.

To investigate the effect of FAK (*PTK2*) knockdown on glycogen synthesis, the radioactivity of cell lysates was measured after the cells were exposed to a glucose tracer in the presence or absence of insulin as previously described [23]. Briefly, cells were subjected to a 4 h serum starvation, then treated with 0, 10 or 120 nmol/l insulin for 30 min. Cells were then exposed to radioactive glucose (d-[U-^14^C]glucose, NEC042B005MC; PerkinElmer) for 90 min before being rinsed twice in ice-cold PBS and frozen at −20°C. Cells were later lysed, glycogen was precipitated and washed, and subsequently dissolved in scintillation fluid for analysis of radioactive content.

### Experimental outcomes

FAK phosphorylation was the primary experimental outcome assessed in all models. Secondary experimental outcomes included glucose transport in the open-muscle biopsy samples, palmitate oxidation or glycogen synthesis in the primary human skeletal muscle cell experiment and phosphorylation status of other proteins in all models.

### Blinding and randomisation

Samples in all experiments were randomly assigned to the treatment conditions indicated. Researchers processing sample lysates were blinded to the group assignment and outcome assessment until the statistical analysis was conducted.

### Inclusion and exclusion criteria

Donors were excluded from participating in the study if they were being treated for diabetes. Primary human skeletal muscle cells were excluded from analysis if they failed to form myotubes upon induction of differentiation. Samples were excluded from analysis only if they were lost or destroyed during sample processing, otherwise all samples were included in the data analysis and interpretation of results.

### Statistical analysis

R base v 3.3.3 (https://cran.r-project.org/bin/windows/base/old/3.3.3/) and open-source packages were used for inferential statistics, while Graphpad Prism v7.02 (La Jolla, CA, USA) was used for generation of figures. When the underlying assumptions were not violated, parametric tests were used to make statistical inferences, otherwise we implemented non-parametric alternatives. Specific omnibus tests are indicated in figure legends. When significant effects were indicated by omnibus testing, post hoc pairwise comparisons were made and adjusted using the Benjamini–Hochberg false discovery rate correction. The threshold for significance (α) was set to 0.05.

## Results

### AMPK activation reduces p-FAK^Y397^

**Fig. 1:**
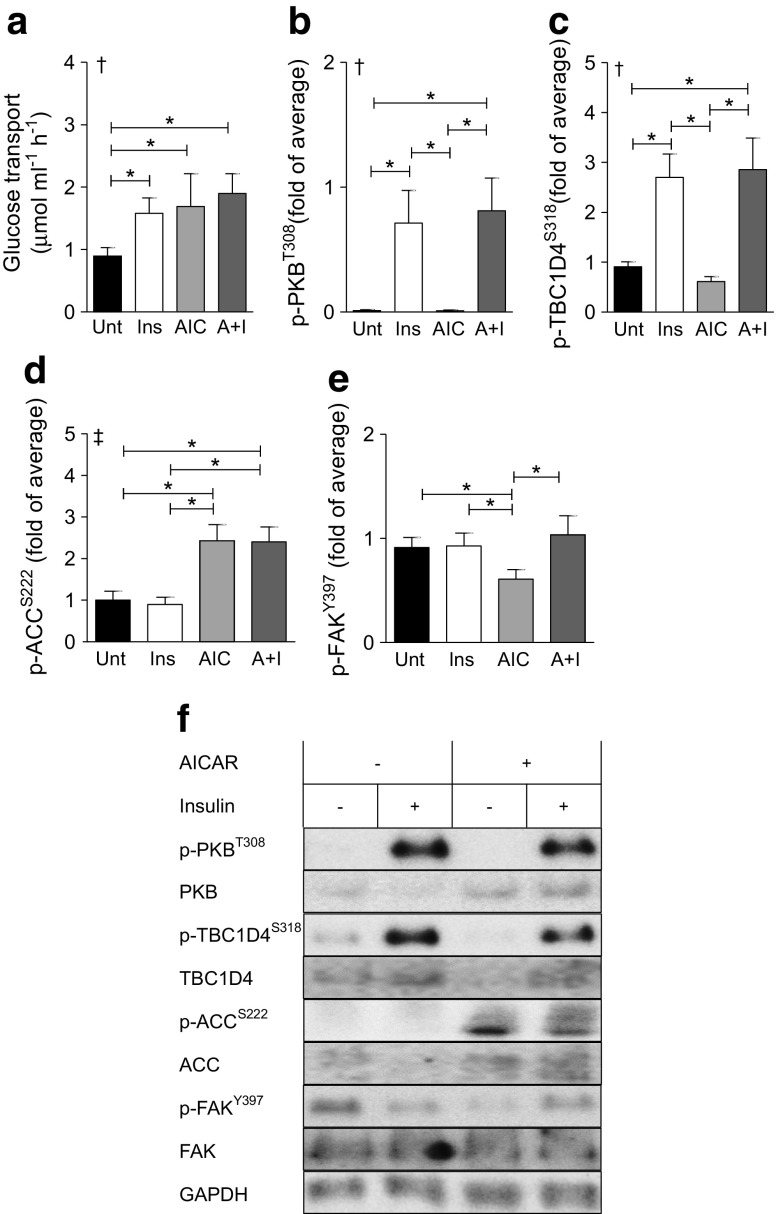
Effect of insulin and AICAR on glucose uptake and signal transduction. Human skeletal muscle strips were incubated in the absence (Unt) or presence of 120 nmol/l insulin (Ins), 2 mmol/l AICAR (AIC), or both (A+I), for 1 h. (**a**) Glucose transport. (**b**) p-PKB^T308^. (**c**) p-TBC1D4^S318^. (**d**) p-ACC^S222^. (**e**) p-FAK^Y397^. (**f**) Representative blots. Results are means ± SEM for matched samples from *n* = 11 participants. The threshold for significance (α) was set to 0.05. *Significant pairwise difference between indicated groups as detected by pairwise post hoc tests after false discovery rate correction. ^†^Significant differences among groups as detected by Friedman’s test (assumptions for two-way repeated measures ANOVA were not met). ^‡^Significant AICAR effect as detected by two-way repeated measures ANOVA, ^§^Significant AICAR-by-insulin interaction as detected by two-way repeated measures ANOVA

Human skeletal muscle strips were incubated in the absence or presence of 120 nmol/l insulin or 2 mmol/l AICAR, or both, to assess the effects on glucose transport and signal transduction (Fig. 1a–f). Insulin and AICAR increased glucose transport in isolated skeletal muscle (Fig. 1a). Insulin increased p-PKB^T308^ and p-TBC1D4^S318^ (Fig. 1b,c), while AICAR increased phosphorylation of acetyl-CoA carboxylase (ACC), a marker of AMPK activation, at S222 (Fig. 1d). AICAR reduced p-FAK^Y397^, whereas insulin had no effect (*p* > 0.89) (Fig. 1e). Glyceraldehyde-3-phosphate dehydrogenase (GAPDH) was used as a loading control and was not affected by insulin or AICAR.

**Fig. 2:**
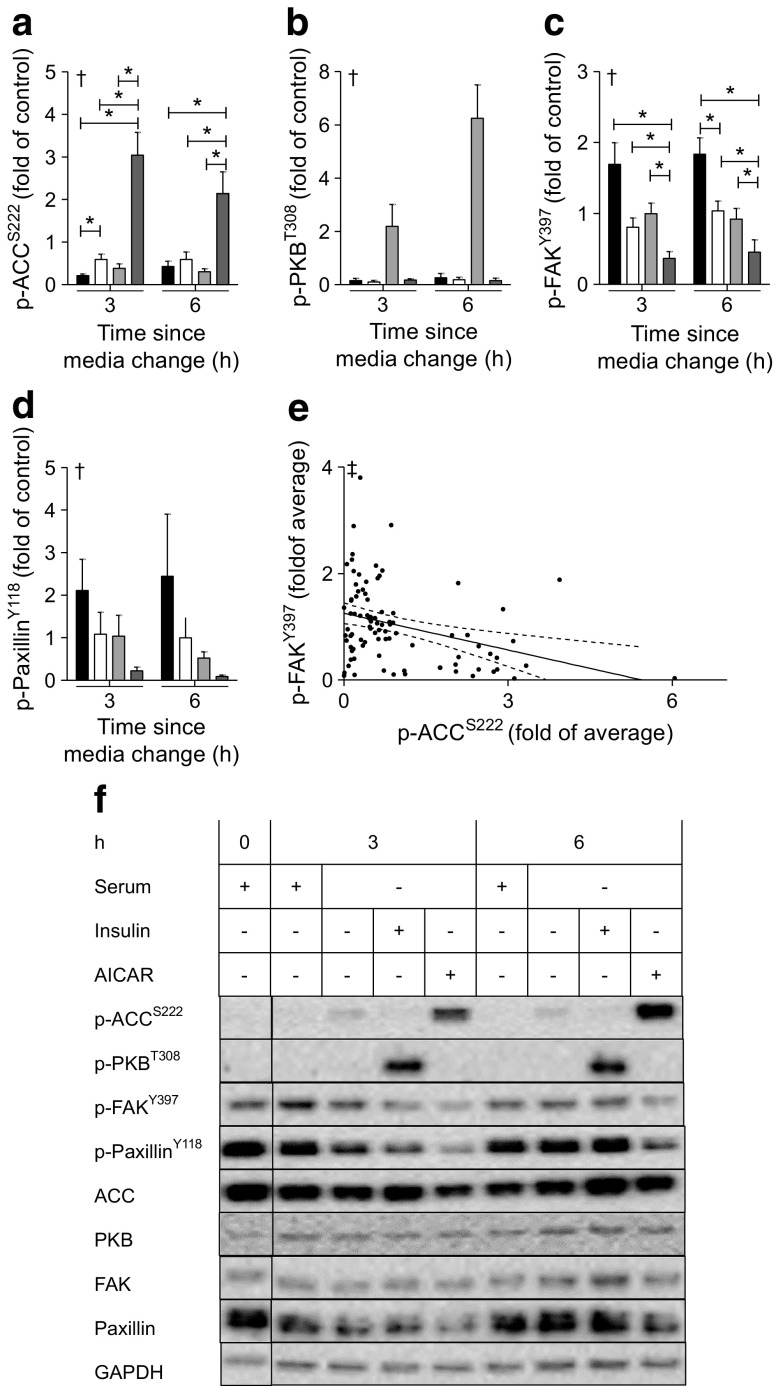
Effect of serum starvation and AICAR treatment on FAK phosphorylation and AMPK activation. Primary human myotubes were incubated for 3–6 h with post-differentiation media (serum; black bars), serum-free post-differentiation media (no serum; white bars), 120 nmol/l insulin (no serum + insulin; light grey bars) or 2 mmol/l AICAR (no serum + AICAR; dark grey bars). Results were compared with untreated myotubes harvested at 0 h (baseline control). (**a**) p-ACC^S222^ (*n* = 10 from matched cultures). (**b**) p-PKB^T308^ (*n* = 4 from matched cultures). (**c**) p-FAK^Y397^ (*n* = 10 from matched cultures). (**d**) p-Paxillin^Y118^ (*n* = 3 from matched cultures). (**e**) Correlation analysis between p-ACC^S222^ and p-FAK^Y397^ (*N* = 90 from 10 matched samples at 9 time-by-treatment levels). (**f**) Representative blots. In (**a**–**d**), results are mean ± SEM. In (**e**), points represent paired data from (**a**, **c**) and the least-squares regression line is plotted with dashed lines to indicate the 95% CI. The threshold for significance (α) was set to 0.05. *Significant pairwise difference between indicated groups as detected by pairwise post hoc tests after false discovery rate correction. ^†^Significant differences among groups as detected by Friedman’s test (assumptions for repeated measures ANOVA were not met). ^‡^Significant correlation as detected by Kendall’s τ (τ = −0.22, assumptions for Pearson’s test were not met)

Differentiated primary human skeletal muscle cells were used for p-FAK determination in the absence or presence of serum, and in response to AICAR or insulin stimulation. AICAR treatment and serum starvation increased p-ACC^S222^, while insulin increased p-PKB^T308^ (Fig. 2a,b). In contrast, p-FAK^Y397^ and the FAK target p-paxillin^Y118^, was lowest in serum-starved AICAR-treated myotubes (Fig. 2c,d). Furthermore, an inverse relationship between p-ACC^S222^ and p-FAK^Y397^ was observed (Fig. 2e). Insulin stimulation did not alter p-FAK^Y397^ (*p* > 0.39) (Fig. 2c). The abundance of total FAK, total ACC, total PKB and total paxillin was unaffected by the length of serum starvation or the treatments given to the cells (Fig. 2f). Even loading was verified by using GAPDH as a control.

### Silencing FAK increases lipid oxidation

**Fig. 3:**
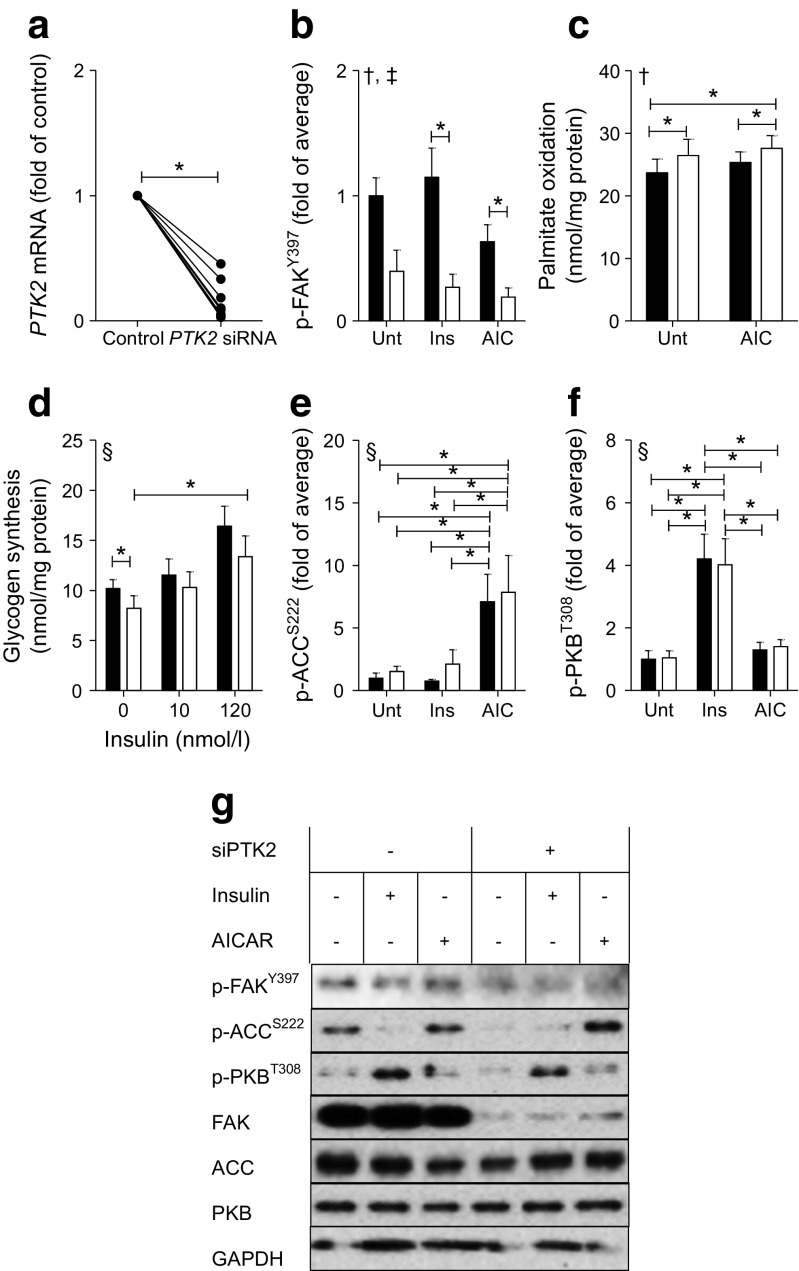
Effect of *PTK2* silencing on palmitate oxidation and glycogen synthesis in skeletal muscle. Primary human skeletal muscle cells were transfected with control siRNA (black bars) or siRNA directed against *PTK2*, the FAK gene (white bars). Cells were harvested for mRNA analysis (**a**) or were untreated (Unt) or treated with 120 nmol/l insulin (Ins) (**b**, **e**, **f**) or 2 mmol/l AICAR (AIC) (**b**, **c**, **e**, **f**). (**a**) *PTK2* mRNA (*n* = 8 from matched cultures). (**b**) p-FAK^Y397^. (**c**) Palmitate oxidation. (**d**) Glycogen synthesis in insulin-treated cells. (**e**) p-ACC^S222^. (**f**) p-PKB^T308^. (**g**) Representative blots. In (**a**) individual responses from all samples are shown. In (**b**–**f**), results are mean ± SEM for matched samples from *n* = 6–8 cultures. The threshold for significance (α) was set to 0.05. *Significant pairwise difference between indicated groups as detected by paired *t* test (**a**) or pairwise post hoc tests after false discovery rate correction (**b**–**f**), ^†^Significant gene-silencing effect as detected by two-way repeated measures ANOVA. ^‡^Significant pharmacological treatment effect as detected by two-way repeated measures ANOVA. ^§^Significant differences among groups as detected by Friedman’s test (assumptions for two-way repeated measures ANOVA were not met)

Transfection of primary human muscle cells with siRNA directed against the FAK gene resulted in more than a 50% reduction of *PTK2* mRNA (Fig. 3a). Gene silencing led to a reduction in total FAK protein and, consequently, p-FAK^Y397^ (Fig. 3b). An increase in palmitate oxidation (Fig. 3c) and a decrease in glycogen synthesis (Fig. 3d) was detected after *PTK2* silencing. AICAR treatment increased p-ACC^S222^ (Fig. 3e). Insulin increased p-PKB^T308^ (Fig. 3f). Total abundance of ACC and PKB was not altered due to gene silencing or treatment with AICAR or insulin (Fig. 3g). GAPDH was used as a loading control and was not affected by insulin, AICAR or *PTK2* silencing.

## Discussion

The appropriate balance between insulin signalling and AMPK activation is critical for maintaining metabolic health. Insulin stimulation and AMPK activation can independently increase GLUT4 translocation and glucose uptake via differential phosphorylation of TBC1D1 and TBC1D4 Rab GTPase-activating (GAP) proteins in skeletal muscle [2, 24, 25]. Here, we confirm our earlier finding that insulin and AICAR increase glucose transport in human skeletal muscle [26]. Using several models to study human skeletal muscle, we provide new evidence that AMPK activation reduces FAK tyrosine phosphorylation. Thus, FAK appears to integrate energy-sensing signals within the cell. AICAR reduced p-FAK^Y397^ in human skeletal muscle. In AICAR-stimulated serum-starved primary human skeletal muscle cells, p-ACC^S222^ is inversely correlated with p-FAK^Y397^. Because FAK activation depends on the sequential phosphorylation of Y397 and Y576/Y577 [11], the reduction in p-FAK^Y397^ in human skeletal muscle, concomitant with the increase in AMPK activity, implies that FAK activity is reduced. Our findings that phosphorylation of paxillin at Y118, a FAK target site, was reduced under conditions that also reduce FAK phosphorylation, further indicate that FAK activity was dampened. While the modulation of FAK activity is multifactorial, our evidence from different paradigms collectively point to an inverse relationship between AMPK and FAK activation.

FAK acts as a sensor of mechanical load and is a constituent of the anabolic signalling pathway in skeletal muscle. Resistance exercise undertaken in the postprandial state increases FAK phosphorylation in human skeletal muscle, concomitant with anabolic signalling [10, 12]. As resistance training increases AMPK signalling in human skeletal muscle [27, 28], particularly if non-habitual resistance exercise is performed [29], our results would predict a decrease in FAK phosphorylation after exercise or AMPK activation. However, our studies were performed in a controlled system whereby many of the myriad effects of exercise are precluded, including hormonal perturbations, altered redox state, force transduction and shifts in calcium signalling. Thus, we propose that AMPK activation negatively regulates FAK signalling.

FAK activation is implicated in insulin signalling and muscle cell differentiation [9, 30]. Therefore, we investigated insulin-mediated regulation of FAK in human skeletal muscle. As insulin increases FAK activity in rodent skeletal muscle [9, 30–32], we expected insulin treatment to increase p-FAK^Y397^. However, FAK phosphorylation was unaltered in both insulin-stimulated human skeletal muscle and primary cultured myotubes. In contrast, we report that silencing the FAK gene, *PTK2*, reduces glycogen synthesis in primary human skeletal muscle cells. Moreover, incubation of rat primary cardiomyocytes with a FAK inhibitor reduces glucose transport [33]. Preliminary experiments reveal counter-regulation of p-FAK^Y397^ due to insulin and AICAR treatment in HEK cells (data not shown). Since the role of FAK in murine neuronal cells is to attenuate insulin signalling, a tissue-specific role for FAK has been proposed [34]. Our observation that FAK knockdown impairs glycogen synthesis despite FAK phosphorylation being unchanged after insulin stimulation indicates an insulin-independent role for FAK in glucose handling. Collectively, these results suggest that insulin-mediated phosphorylation of FAK is tissue and species specific. Our findings are particularly relevant since we describe the impact of AMPK activation and insulin on FAK phosphorylation in human skeletal muscle for the first time.

We explored the metabolic consequences of silencing the FAK gene, *PTK2*, in human skeletal muscle. Increased IL-8, secreted from primary human skeletal myotubes derived from individuals with type 2 diabetes into conditioned culture media, upregulates FAK signalling in skeletal muscle, suggesting a role for FAK in insulin resistance [35]. Using siRNA against *PTK2*, we show that reducing FAK protein abundance increases palmitate oxidation in human skeletal muscle. In rat cardiomyocytes, siRNA-mediated silencing of *PTK2* reduces oligomycin-induced glucose transport [33]. Collectively, these data indicate that a reduction in FAK protein abundance shifts the metabolic programming of skeletal and cardiac muscle to favour lipid oxidation. Though beyond the scope of this study, future research could elucidate the mechanism by which FAK mediates lipid oxidation by utilising FAK inhibitors or *PTK2*-silenced myotubes treated specifically with malonyl-CoA or C75, a fatty acid synthase inhibitor. Though it remains to be determined whether FAK activation impairs lipid oxidation, our findings imply that FAK has a role in the control of metabolic substrate utilisation in human skeletal muscle.

Inhibition of FAK activity may have therapeutic benefits for the treatment of various chronic disease states. Because diabetes is characterised by impaired lipid oxidation in skeletal muscle [36], FAK inhibition may enhance metabolic flexibility. FAK activity correlates with cancer progression by promoting cell survival, proliferation and migration [37], and FAK inhibitors are actively being investigated to treat cancer [38]. Inhibition of FAK may improve clinical outcomes for metabolic disorders or cancer, since FAK is an effector of insulin-like growth factor 1 [39, 40]. As cancer cells tend to favour glycolytic metabolism (the ‘Warburg effect’) [41, 42], and our data implicate FAK as a moderator of lipid oxidation, the efficacy of FAK inhibitors in cancer may be attributed to substrate shifts and increased lipid oxidation relative to glucose oxidation. Importantly, the use of FAK inhibitors may not be equally effective across the lifespan, since aged skeletal muscle is characterised by nuclear localisation of the FAK protein and reduced responsiveness to FAK inhibitors [15]. Skeletal muscle from lean and obese individuals differ by approximately 30% when it comes to the percentage of energy demands being met by lipid oxidation [43]. In this context, the ~ 10% increase in palmitate oxidation in primary skeletal muscle cells due to *PTK2* silencing is clinically relevant, especially if FAK impairs glucose uptake, as may be suggested by our finding that glycogen synthesis is enhanced after *PTK2* silencing. Further studies are warranted to validate FAK inhibition as a strategy for the treatment of lipid metabolism disorders.

Using AICAR-treated human skeletal muscle biopsies and primary human skeletal muscle cells subjected to serum starvation or AICAR treatment, we provide evidence that AMPK activity reduces FAK tyrosine phosphorylation. In primary human skeletal muscle cells, *PTK2* silencing increased palmitate oxidation, indicating that FAK functions as an inhibitor of lipid oxidation. AMPK activity may reduce FAK tyrosine phosphorylation and FAK activity via several pathways. AMPK may activate a phosphatase to remove tyrosine phosphorylation on FAK. Since both protein phosphatase 2A and dual-specificity phosphatase (DUSP) are activated by AMPK, they are natural candidates [44, 45]. The use of a FAK inhibitor in glioma cells increases expression of *DUSP1* and *DUSP5*, suggesting the existence of a negative feedback loop [46]. Alternatively, AMPK activity may antagonise FAK signalling by potentiating FAK-related non-kinase competition for FAK binding partners. AMPK may sequester FAK away from the cell membrane, thereby preventing its autophosphorylation and activation. This is supported by the fact that resveratrol-induced AMPK activation leads to cytosolic localisation of the four-point-one, ezrin, radixin, moesin (FERM) domain of FAK, which inhibits Y397 autophosphorylation [47]. An AMPK-mediated reduction in FAK activity may increase lipid oxidation in skeletal muscle and effectively attenuate cancerous phenotypes in non-muscle tissue.

In conclusion, AMPK activation suppresses FAK tyrosine phosphorylation in human skeletal muscle. Silencing of the FAK gene (*PTK2*) increases lipid oxidation in skeletal muscle. Collectively, our results implicate an AMPK–FAK relationship in skeletal muscle. While cell-specific differences in the regulation of FAK due to AMPK activation and insulin signalling are likely to exist, treatment strategies for metabolic disorders may be further validated through the study of the AMPK–FAK relationship in skeletal muscle, hepatic tissue or adipose tissue. Furthermore, a better understanding of the opposing influences of AMPK and insulin signalling on FAK in other tissues may provide insight into oncogenic processes. Elucidation of the interaction of AMPK and insulin signalling, and their roles in FAK regulation, may lead to novel therapeutic strategies for chronic diseases as seemingly disparate as type 2 diabetes and cancer.

## Electronic supplementary material

ESM Tables (PDF 93 kb)

## Abbreviations

ACC: Acetyl-CoA carboxylase
AICAR: 5-aminoimadazole-4-carboxamide ribonucleotide
AMPK: AMP-activated protein kinase
FAK: Focal adhesion kinase (also known as protein kinase 2)
GAPDH: Glyceraldehyde-3-phosphate dehydrogenase
PKB: Protein kinase B (also known as Akt)
TBC1D1: TBC1 domain family member 1
TBC1D4: TBC1 domain family member 4 (also known as Akt substrate of 160 kDa, AS160)
TBS: TRIS-buffered saline
TBST: TRIS-buffered saline with Tween-20
