# Supplementary material for: FAK tyrosine phosphorylation is regulated by AMPK and controls metabolism in human skeletal muscle
Source: Diabetologia. 2017 Oct 11;61(2):424–32. doi: 10.1007/s00125-017-4451-8 (PMC6449061; doi:10.1007/s00125-017-4451-8)
Supplement: Supplementary file 1 — (PDF 93 kb) [file 125_2017_4451_MOESM1_ESM.pdf]

Online Supplemental Materials

ESM Table 1:

List of Antibodies used for Western-Blot Analysis

Target	Vendor	Product Number
p-FAK^{Y397}	Cell Signaling (Danvers, MA)	3283
FAK total	Cell Signaling	3285
p-ACC^{S222}	Cell Signaling	3661
ACC total	Cell Signaling	3676
Paxillin total	Cell Signaling	2542
p-Paxillin^{Y118}	Cell Signaling	2541
p-TBC1D4^{S318}	Cell Signaling	8619
TBC1D4	Merck Millipore (Darmstadt, Germany)	07-741
p-PKB^{T308}	Cell Signaling	4056
PKB total	Cell Signaling	9272
GAPDH	Santa Cruz Biotechnology (Dallas, TX)	sc-257758

ESM Table 2:

Oligonucleotides used in the qPCR Analysis

Gene	Exons	Forward Primer	Reverse Primer
PTK2	29-31	CCTGGACCGGTCGAATGATA	TGCCATCTCAATCTCTCGGT
PPIB	2-3	ATGTAGGCCGGGTGATCTTT	CATCTCCCCTGGTGAAGTCT
TBP	3-5	TTCGGAGAGTTCTGGGATTG	GAAAATCAGTGCCGTGGTTC
B2M	2-4	TGTCTTTCAGCAAGGACTGG	AGCAAGCAAGCAGAATTTGG
TFRC	15-16	AATGCTGCTTTCCCTTTCCT	TCCATGGTGGTACCCAAATAA
